# Supplementary material for: Identification of CD44 as a Reliable Biomarker for Glioblastoma Invasion: Based on Magnetic Resonance Imaging and Spectroscopic Analysis of 5-Aminolevulinic Acid Fluorescence
Source: Biomedicines. 2023 Aug 24;11(9):2369. doi: 10.3390/biomedicines11092369 (PMC10525185; doi:10.3390/biomedicines11092369)
Supplement: Supplementary file 1 [file biomedicines-11-02369-s001.zip › Supplementary Table S1.pdf]

Supplementary Table S1. Characteristics of 50 patients with GBM, including 21 patients who were also enrolled in the study of 5-ALA spectroscopy.

No. (All)	No. (ALA)	Age (years)	Sex	KPS (%)	mMGMT	EOR	PFS (M)	OS (M)	Outcome	Type on MRI	CD44 (P/C ratio)	CD44 (C)	CD44 (P)
**1**		79	M	70	+	GTR	9	18	D	H	32.8	0.98	32.13
**2**		75	M	60	-	GTR	11	14	D	H	18	1.5	27
**3**		44	M	90	-	GTR	18.1	55	D	L	0.85	14.3	12.1
**4**		80	M	60	-	PR	1.3	9	D	L	0.27	29.6	8.2
**5**		53	M	80	+	GTR	2.1	13	D	H	3.2	2.16	6.95
**6**		69	M	90	-	PR	1.3	28	D	L	1.03	20.9	21.5
**7**		53	F	90	+	GTR	5.9	8	D	H	3.2	1.3	4.2
**8**		64	M	70	-	GTR	7.3	16	D	L	3.2	3.3	10.6
**9**		64	M	70	-	PR	2.9	14	D	H	13.05	0.3	3.9
**10**		60	M	70	+	PR	3.7	20	D	H	7.3	7.19	52.3
**11**		52	F	70	-	GTR	8.5	46+	A	L	1.4	1.57	2.2
**12**		76	M	70	+	GTR	1	25	D	L	1.2	17.9	14.9
**13**		74	M	90	-	GTR	15.9	21	D	H	19.1	1.2	22.8
**14**		77	M	90	-	GTR	6.4	19	D	H	0.15	0.27	0.04
**15**		72	M	90	-	GTR	15.7	34	D	L	1.19	0.009	0.011
**16**		58	F	90	+	GTR	13.2	17+	A	L	2.55	1.16	2.95
**17**		37	M	90	-	PR	1.4	14+	A	H	9.84	1.33	13.1
**18**		30	M	70	-	GTR	9.6	24	D	L	0.8	2.8	2.1
**19**		60	M	70	-	PR	3.7	20.5	D	H	8.21	7.2	59.1
**20**		63	F	60	+	GTR	24.1	31.6	D	H	8.38	4.9	40.8
**21**		76	M	90	-	GTR	6.4	18.6	D	L	0.15	0.3	0.04
**22**		85	M	80	+	GTR	32.9+	32.9+	A	L	4.71	1.5	7.1
**25**		53	M	80	+	GTR	2.1	13.1	D	L	5.38	2.2	11.6
**26**		64	M	70	+	GTR	7.3	16.3	D	H	9.38	3.3	31.1
**27**		19	M	60	-	PR	16	16	D	H	0.86	20.9	18
**33**		79	F	60	-	PR	1.3	25+	A	L	1.06	0.3	0.3
**34**		76	F	90	+	PR	20.6+	20.6+	A	H	9.84	1.3	13.1
**38**		80	M	100	-	GTR	15.8+	15.8+	A	L	2.37	0.3	0.6
**39**		73	M	80	+	PR	8.5	8.5	D	H	13.08	3.2	41.7
**23**	**A1**	63	F	80	-	GTR	46	59	D	L	1.3	55.2	69.1
**24**	**A2**	62	M	70	-	STR	14	17	D	H	14.1	0.85	12.02
**28**	**A3**	66	M	90	+	GTR	7	16	D	H	18.4	1.75	32.1
**29**	**A4**	53	M	80	+	GTR	9	33	D	H	9.8	5.86	57.5
**30**	**A5**	61	F	80	+	GTR	8	26	D	L	0.65	19.9	13
**31**	**A6**	69	M	70	-	PR	7	10	D	H	46.5	0.4	18.6
**32**	**A7**	67	M	70	+	GTR	8.2	12.1	D	H	15.9	1	16.3
**35**	**A8**	64	F	90	-	GTR	80+	80+	A	L	1.9	2.7	5.3
**36**	**A9**	71	M	90	-	GTR	6	9.7	D	H	16.8	4.1	69.1
**37**	**A10**	79	F	70	-	PR	29	43.3	D	L	0.8	2.8	2.1
**40**	**A11**	65	F	70	+	GTR	73+	73+	A	L	1	2.9	2.8
**41**	**A12**	67	M	60	+	STR	3.8	6.8	D	H	8.1	1.6	13
**42**	**A13**	86	M	80	+	GTR	2.4	2.4	D	H	7.5	4.1	30.8
**43**	**A14**	71	F	80	-	GTR	4.8	6.8	D	H	8.1	3.1	25
**44**	**A15**	65	M	70	+	STR	9	23.8	D	L	0.9	3.3	3.1
**45**	**A16**	66	M	70	-	GTR	3.9	5.1	D	H	10.3	4	41.2
**46**	**A17**	59	M	70	+	PR	2.6	8.3	D	H	9.2	1.6	14.8
**47**	**A18**	64	F	80	+	STR	7.4	23.5	D	H	8.4	3.2	27
**48**	**A19**	72	M	90	-	GTR	15.7	26.5+	A	L	2.4	1	2.4
**49**	**A20**	73	M	70	-	STR	12	24	D	L	0.03	29.8	0.7
**50**	**A21**	78	M	60	-	PR	4	6	D	H	15.5	4	61.2

GBM, glioblastoma multiforme

5-ALA, 5-aminolevulinic acid

No. (all), number of all 50 patients

No. (ALA), number of 21 patients enrolled in the 5-ALA spectroscopic study

M, male; F, female

KPS, Karnofsky performance status

mMGMT, methylation of the O6-methylguanine-DNA methyltransferase

EOR, extent of resection

GTR, gross total resection; STR, subtotal resection; PR, partial resection

PFS, progression-free survival

OS, overall survival

D, dead; A, alive

H, high invasive type; L, low invasive type

P/C ratio, periphery/core ratio

C, tumor core

P, tumor periphery
