# Supplementary material for: Identification of CD44 as a Reliable Biomarker for Glioblastoma Invasion: Based on Magnetic Resonance Imaging and Spectroscopic Analysis of 5-Aminolevulinic Acid Fluorescence
Source: Biomedicines. 2023 Aug 24;11(9):2369. doi: 10.3390/biomedicines11092369 (PMC10525185; doi:10.3390/biomedicines11092369)
Supplement: Supplementary file 1 [file biomedicines-11-02369-s001.zip › Supplymentary Figures S1 and S2.pdf]

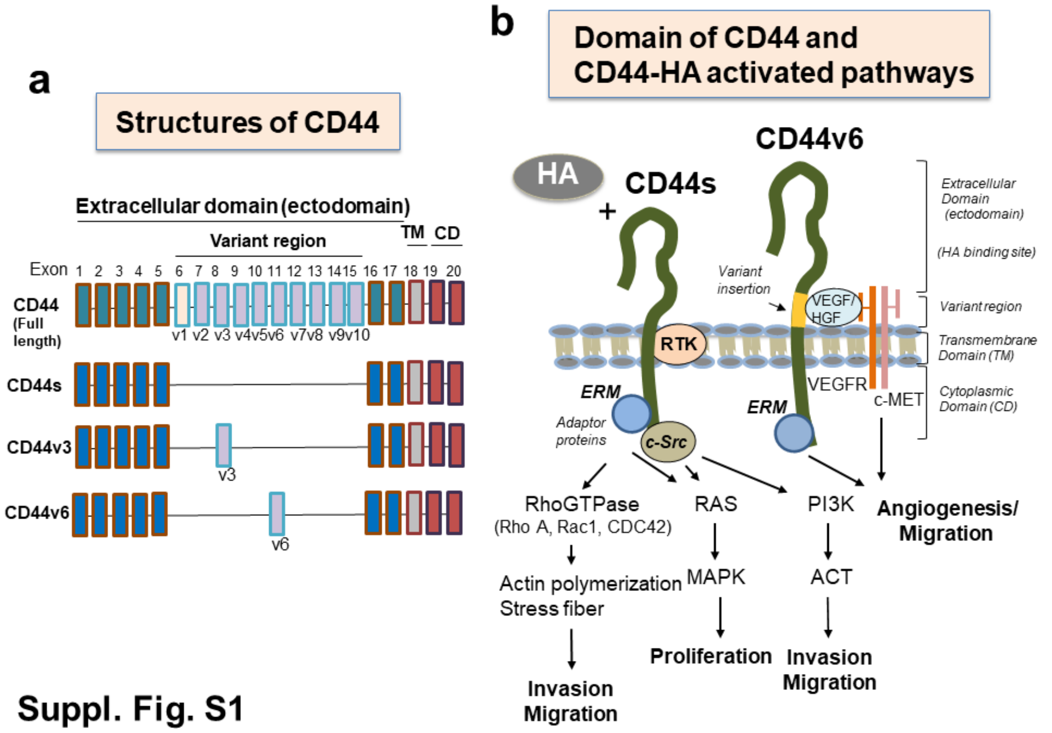

Suppl. Fig. S1

Supplementary Figure S1. Structures and functions of CD44. a) The CD44 gene encodes 20 exons, of which exons 6 to 15 are alternatively spliced and inserted into the variant region of CD44 as variable exons (v1-v10). The figure presents gene structures of CD44 standard isoform (CD44s) and CD44 variant isoforms (CD44v3 and CD44v6). b) Domain structures of CD44 molecule and signaling pathways activated by the interaction of CD44 and hyaluronic acid (HA). CD44 consists of the following three domains: the extracellular domain (ectodomain), the transmembrane domain, and the cytoplasmic domain. The extracellular domain includes HA binding site and the variant region where variant exons are inserted, thus providing activity as a co-receptor for various growth factors and cytokines. The transmembrane domain activates receptor tyrosine kinase (RTK) and elevates the activities of non-receptor kinases of Src family. The intracellular signaling pathways enhance the activity of the downstream pathways such as mitogen-activated protein kinase (MAPK) and phosphoinositide 3-kinase (PI3K), thus promoting cellular processes including migration, invasion, proliferation, and angiogenesis. The cytoplasmic domain is released by cleavage of the transmembrane domain. The released ICD fragments translocate into the nucleus and activate various genes as transcription factor. CD44v6 has a binding site for hepatocyte growth factor (HGF) and vascular endothelial growth factor (VEGF). When VEGF binds to the co-receptor in the variant region, VEGF is enhanced to bind to its receptor VEGFR, resulting in promoting angiogenesis. At this time, the activity of c-MET, a receptor for HGF, is inhibited by forming a heterodimer with VEGFR. ERM: Ezrin/Radixin/Moesin.

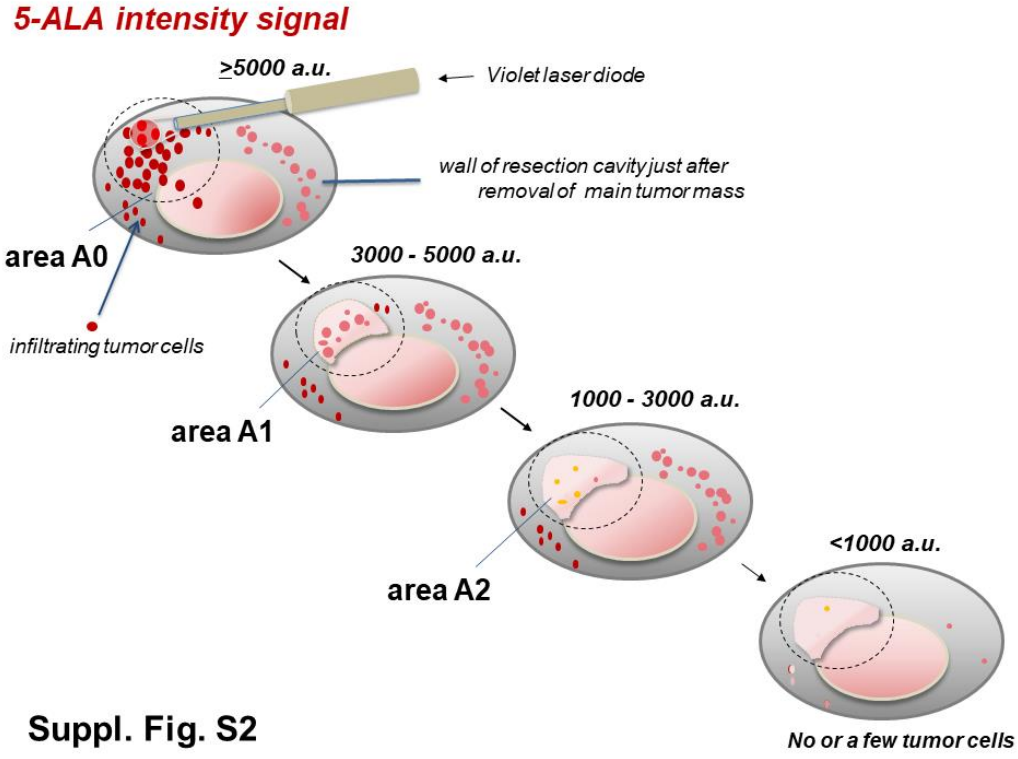

Suppl. Fig. S2

Supplementary Figure S2. Illustration showing surgical procedures for tumor resection in the peritumoral area under guidance of spectroscopic 5-ALA fluorescence.

In the wall of the tumor resection cavity at the tumor border (area A0), many tumor cells remain, presenting high 5-ALA fluorescence intensity ≥5000 a.u. Resection of tumor in this area showing high 5-ALA intensity reveals less tumor cells presenting moderate fluorescence intensity of 3000–5000 a.u. (area A1). Further resection of tumor cells reaches much fewer tumor cells showing fluorescence intensity of 1000–3000 a.u. (area A2). When 5-ALA fluorescence presents an intensity signal <1000 a.u., tumor resection is regarded as finished.
